# Single-Sided Wood Moisture Nondestructive Testing Using Free-Space Microwave Reflection

**DOI:** 10.3390/s26217020

**Published:** 2026-11-06

**Authors:** Jiaoyang Li, Zhao Liu, Xingliang Zhao, Guanyu Piao

**Affiliations:** 1 School of Mechatronic Engineering and Automation, Shanghai University, Shanghai 200444, China; 2 Key Laboratory of Silicate Cultural Relics Conservation, Shanghai University, Ministry of Education, Shanghai 200444, China

**Keywords:** wood moisture, microwave nondestructive testing, free-space reflection, valley-depth feature, wooden cultural relics

## Abstract

Moisture-induced deformation, cracking, and biological deterioration threaten wooden cultural heritage, making reliable, nondestructive moisture assessment essential for preventive conservation. The microwave method has been recognized as an emerging method with advantages of noncontact, fast and high accuracy. However, conventional microwave transmission-based moisture detection methods require access to both sides of a specimen, hindering the inspection of fixed components with inaccessible rear surfaces. This study investigates single-sided, non-contact wood moisture assessment using the principle of free-space microwave reflection. A three-dimensional full-wave antenna–wood model identified significant moisture-dependent variations in a valley near 1.1 GHz and its adjacent peak. Their magnitude difference was defined as a valley-depth feature, with an optimized 100 mm standoff selected to balance linearity and sensitivity. The sensing system was developed based on the optimized experimental parameters from the simulation. And the calibration test showed a linear relationship between valley depth and relative permittivity (*R*^2^ = 0.98). Pine and paulownia specimens below 30% moisture content were measured in two polarization orientations, yielding short-term repeatability standard deviations less than 0.013 dB. Independent validation achieved the lowest errors for paulownia with the electric field perpendicular to the grain, with RMSE = 1.27% and MAE = 0.99%. An in-situ trial on a historic wooden door demonstrated field deployment feasibility and measurable spatial and temporal variations in the reflection response. These findings support the potential of the method for single-sided, non-contact wood moisture assessment.

## 1. Introduction

Wooden cultural heritage objects constitute an irreplaceable component of the global cultural legacy, preserving valuable historical, artistic, technological, and scientific information. Nevertheless, their long-term preservation is strongly influenced by environmental moisture because wood is a porous and hygroscopic material that continuously exchanges water with the surrounding atmosphere. Depending on the moisture state, water may be present as bound water within the cell walls or as water vapor and free liquid water within the cell lumina and other macroscopic voids [1]. Environmental moisture changes initially affect exposed surfaces, and moisture subsequently redistributes toward the interior, producing transient spatial gradients in moisture content. These gradients induce differential shrinkage and swelling, while the orthotropic and anisotropic nature of wood results in distinct dimensional responses in the radial and tangential directions. The resulting incompatibility of deformation generates internal hygro-mechanical stresses, which may initiate and propagate cracks when the tensile resistance perpendicular to the grain is exceeded, thereby reducing the structural integrity of wooden objects [2,3]. Repeated freeze–thaw exposure may further aggravate deterioration in moisture-containing wood by promoting microstructural cracking and reducing its strength, stiffness, and deformation capacity [4]. In addition, elevated moisture content, together with suitable temperature conditions, facilitates the growth and metabolic activity of wood-decaying fungi, accelerating the degradation of cell-wall constituents and shortening the service life of wooden components [5]. Consequently, variations in moisture content represent a critical factor governing the dimensional stability, mechanical integrity, biological durability, and long-term conservation of wooden cultural heritage.

Accurate determination of wood moisture content is essential for evaluating moisture-induced dimensional deformation, cracking risk, and long-term material deterioration [6]. Existing measurement techniques can generally be classified into direct and indirect methods according to whether moisture content is obtained from the actual mass of water in the specimen or inferred from a moisture-sensitive physical property [7,8]. Among the direct methods, oven drying is the most widely accepted technique and is commonly employed as the reference method for calibrating and evaluating other moisture measurement approaches [9]. In this gravimetric method, moisture content is calculated from the difference between the initial mass of a specimen and its oven-dry mass. High measurement accuracy can generally be achieved when the drying temperature, specimen preparation, weighing procedure, and constant-mass criterion are controlled in accordance with standardized procedures, such as GB/T 1931-2009 [10].

Nevertheless, the method requires the removal and irreversible drying of representative specimens and is therefore destructive. Its relatively long measurement cycle also prevents real-time or continuous monitoring. These limitations make oven drying unsuitable for routine in situ examination of valuable wooden cultural heritage objects. Consequently, indirect techniques are particularly important in heritage conservation because they estimate moisture content with limited or no alteration of the original material. Such methods exploit changes in the electrical, dielectric, acoustic, magnetic, optical, or electromagnetic properties of wood caused by moisture variation. Representative approaches include capacitive or dielectric sensing [11,12], ultrasonic testing [13], nuclear magnetic resonance and magnetic resonance imaging [14], near-infrared spectroscopy [15,16], microwave testing [17,18], and electrical resistance measurement. Among these techniques, the electrical resistance method has been extensively used because of its simple instrumentation, rapid response, and relatively low cost. Below the fibre saturation point (FSP), increasing bound-water content markedly enhances ionic conduction within the cell walls, causing wood resistance to decrease by several orders of magnitude and enabling moisture content to be estimated using empirical resistance–moisture calibration relationships. Wu et al. developed a resistance-based instrument for depth-resolved wood moisture measurement and reported a measurement error of less than 1% under the investigated conditions [19]. However, the method required electrodes to be inserted into the wood, resulting in localized surface damage and making it only minimally invasive. To avoid electrode penetration, Suarez et al. proposed a plate-electrode system for measuring the bulk electrical resistivity of spruce wood and optimized the electrode–wood contact conditions to achieve non-invasive measurements [20]. Despite these developments, conventional resistance meters remain most effective below the FSP. Once free water begins to accumulate within the cell lumina, further increases in moisture content produce comparatively small and less stable changes in electrical resistance, while the measurement becomes more susceptible to wood species, anatomical direction, temperature, moisture distribution, and electrode-contact conditions. To extend electrical measurement above the FSP, Tamme et al. introduced a chrono-potentiometric method based on the electrical charging and discharging behavior of wet wood. For the tested hardwood species, this model improved the estimated single-measurement precision from approximately ±10% for the conventional resistance model to ±1.75% [21]. Although this method represents substantial progress in high-moisture measurement, further validation across a wider range of wood species, moisture distributions, temperatures, and practical conservation environments is required before it can be routinely applied to wooden cultural heritage objects.

Microwave techniques have considerable potential for wood moisture assessment because they enable rapid, nondestructive, and non-contact measurements while retaining sensitivity to the bulk electromagnetic properties of the inspected material [22,23,24]. Their physical basis is the pronounced difference between the dielectric properties of water and those of dry wood. As moisture is absorbed into the cell walls or accumulates within the cell lumina, the effective complex permittivity of wood changes accordingly, thereby modifying the propagation velocity, attenuation, phase, polarization, and reflection of the incident microwave field. These moisture-dependent electromagnetic responses can consequently be used as measurable features for quantitative wood moisture evaluation. The absence of direct mechanical contact is particularly advantageous for wooden cultural heritage objects, for which sampling, electrode insertion, or sustained probe pressure may be unacceptable. Microwave sensing of wood moisture is an established research area. Early investigations demonstrated that microwave attenuation, phase shift, and depolarization depend on moisture content, wood density, and grain orientation [25]. Subsequent free-space microwave studies examined reflection and transmission characteristics and addressed measurement errors associated with multiple reflections during timber inspection [26]. Accordingly, the present study builds on this established sensing principle by evaluating a local peak-to-valley reflection feature and investigating its applicability to single-sided moisture assessment of wooden cultural heritage.

A substantial proportion of previous microwave studies on solid wood have employed transmission or through-material scanning configurations under controlled laboratory conditions. In these systems, the specimen is positioned between the transmitting and receiving components, and its moisture content is estimated from the attenuation and phase shift introduced during microwave propagation [27,28,29]. Because wood is electromagnetically orthotropic, the measured response also depends strongly on the orientation of the electric field relative to the grain. Modern free-space transmission systems therefore commonly use dual-linearly polarized measurements, from which the propagation components parallel and perpendicular to the grain can be separated or combined to reduce errors associated with unknown grain orientation. Calibration, multivariate regression, or inversion models are subsequently established between the measured transmission parameters and reference moisture values [30,31]. Previous investigations have demonstrated that such measurements can provide quantitative estimates of average moisture content and, in some cases, its spatial distribution within sawn timber. Despite these advantages, transmission-based systems present practical limitations for the in-situ examination of wooden cultural heritage. First, the transmitting and receiving components must be placed on opposite sides of the inspected object, which may be impossible for immovable structures, wall-integrated timber members, decorated panels, or objects whose rear surfaces are inaccessible. The associated two-sided arrangement also increases the required installation space and makes antenna alignment more demanding. Second, the transmitted field decreases exponentially with propagation distance in a lossy dielectric. Consequently, increases in specimen thickness, moisture content, and dielectric loss can substantially attenuate the received signal, potentially reducing the signal-to-noise ratio and measurement stability. Transmission measurements are also affected by multiple reflections at the air–wood interfaces, diffraction from specimen edges, wood heterogeneity, and polarization-dependent propagation, all of which require careful calibration or signal-processing procedures. Reflection-based electromagnetic measurements provide a possible means of relaxing these geometric restrictions. Previous studies using open-ended microwave probes, ground-penetrating radar, and antenna-reflection measurements have demonstrated that wood moisture variation can be correlated with changes in permittivity or reflection response [32,33]. However, these investigations have primarily involved contacting or locally coupled probes, radar measurements, or particulate wood materials, whereas broadband free-space reflection measurements of solid wood remain comparatively limited. In a free-space reflection configuration, both microwave transmission and reception can be implemented from the same accessible side of the specimen, eliminating the need for rear-surface access and for a detectable signal to propagate completely through the component. Although the effective penetration depth remains dependent on frequency, moisture level, and wood dielectric loss, this one-sided arrangement is more compatible with the spatial and conservation constraints encountered during in situ heritage inspection. Therefore, the present study investigates a quantitative wood moisture assessment method based on the frequency-dependent free-space microwave reflection response. Moisture-sensitive features are extracted from the measured reflection spectra and correlated with reference moisture contents through a calibration model. The proposed method is subsequently validated experimentally, with the objective of developing a rapid, non-contact, and readily deployable technique for the in-situ moisture assessment of wooden cultural heritage.

The rest of this paper is organized as follows. Section 2 presents the theoretical basis of free-space microwave reflection from moisture-containing wood. Section 3 describes the electromagnetic simulations and the extraction and optimization of the moisture-sensitive reflection feature. Section 4 introduces the specimens, measurement system, experimental procedures, experimental results, quantitative moisture prediction performance and in-situ testing. Section 5 and Section 6 discuss and summarize the main findings of this study.

## 2. Principle

### 2.1. Microwave Dielectric Response of Moist Wood

Microwave-based moisture detection relies on the sensitivity of the dielectric properties of wood to its moisture content. The complex permittivity of wood can be expressed as:

*ε* = *ε*′ − *jε*″ (1)

where *ε*′ is the real part of the complex permittivity and describes the material’s ability to store electric-field energy, whereas *ε*″ is the imaginary part and accounts for dielectric loss caused by delayed polarization. Water molecules have a large permanent dipole moment and therefore undergo orientational polarization in an alternating electric field. At microwave frequencies, molecular reorientation does not follow the rapidly varying field instantaneously, producing a phase lag between the polarization and the applied field. Moist wood consequently exhibits stronger dielectric energy-storage and loss responses than dry wood.

Wood is a porous, heterogeneous, and anisotropic material composed mainly of cell-wall substances, air, and water. Because liquid water has a substantially higher microwave permittivity than oven-dry wood, increasing the moisture content generally increases both the real and imaginary parts of the effective complex permittivity. Below the fiber saturation point, water is present predominantly as bound water in the cell walls. In this range, moist wood may be approximated as a composite consisting of an air phase and a solid-solution phase formed by cell-wall material and bound water. Effective-medium theory therefore relates the macroscopic permittivity of wood to the permittivities, volume fractions, and spatial distributions of these constituent phases.

The effective dielectric response also depends on wood species, density, grain orientation, temperature, and measurement frequency. The preferential alignment of cells along the grain produces different polarization responses parallel and perpendicular to the grain. Thus, the measured free-space reflection contains moisture-dependent information together with contributions from species-specific dielectric properties and wood anisotropy.

Accordingly, within the scope of this study, the effective complex permittivity may be represented by the general functional relationship:

*ε*_eff_ = *G_ε_*(MC,*ρ*,*θ*,*f*,*T*) (2)

where MC is the moisture content, *ρ* is the wood density, *θ* is the angle between the electric-field polarization and the grain direction, *f* is the measurement frequency, *T* is the temperature, and *G_ε_* denotes an effective dielectric mapping determined by the multiphase composition and microstructure of wood. Because wood is intrinsically heterogeneous and anisotropic, this mapping cannot generally be represented accurately by a single closed-form expression.

Various effective-medium models have been developed to describe the relationship between wood moisture content and effective complex permittivity. Effective-medium models, such as the Maxwell–Garnett and Bruggeman models, have been widely used to estimate the effective dielectric properties of heterogeneous materials based on the dielectric properties and volume fractions of their constituent phases [34]. Paz et al. [35] investigated dielectric mixing models for woody biomass and found that the Maxwell–Garnett model provided good agreement with the measured dielectric properties under the investigated conditions. Furthermore, Rönnow et al. [36] compared Bruggeman and Maxwell–Garnett models for conifer wood chips and reported that both approaches agreed reasonably well with experimental results below the fiber saturation point. Their measurements also demonstrated that the real and imaginary components of the complex permittivity generally increased with moisture content. These findings provide a basis for representing moisture-dependent dielectric variations in wood, although the quantitative relationship depends on material properties and measurement conditions.

### 2.2. Free-Space Reflection from a Finite-Thickness Wood Plate

**Figure 1:**
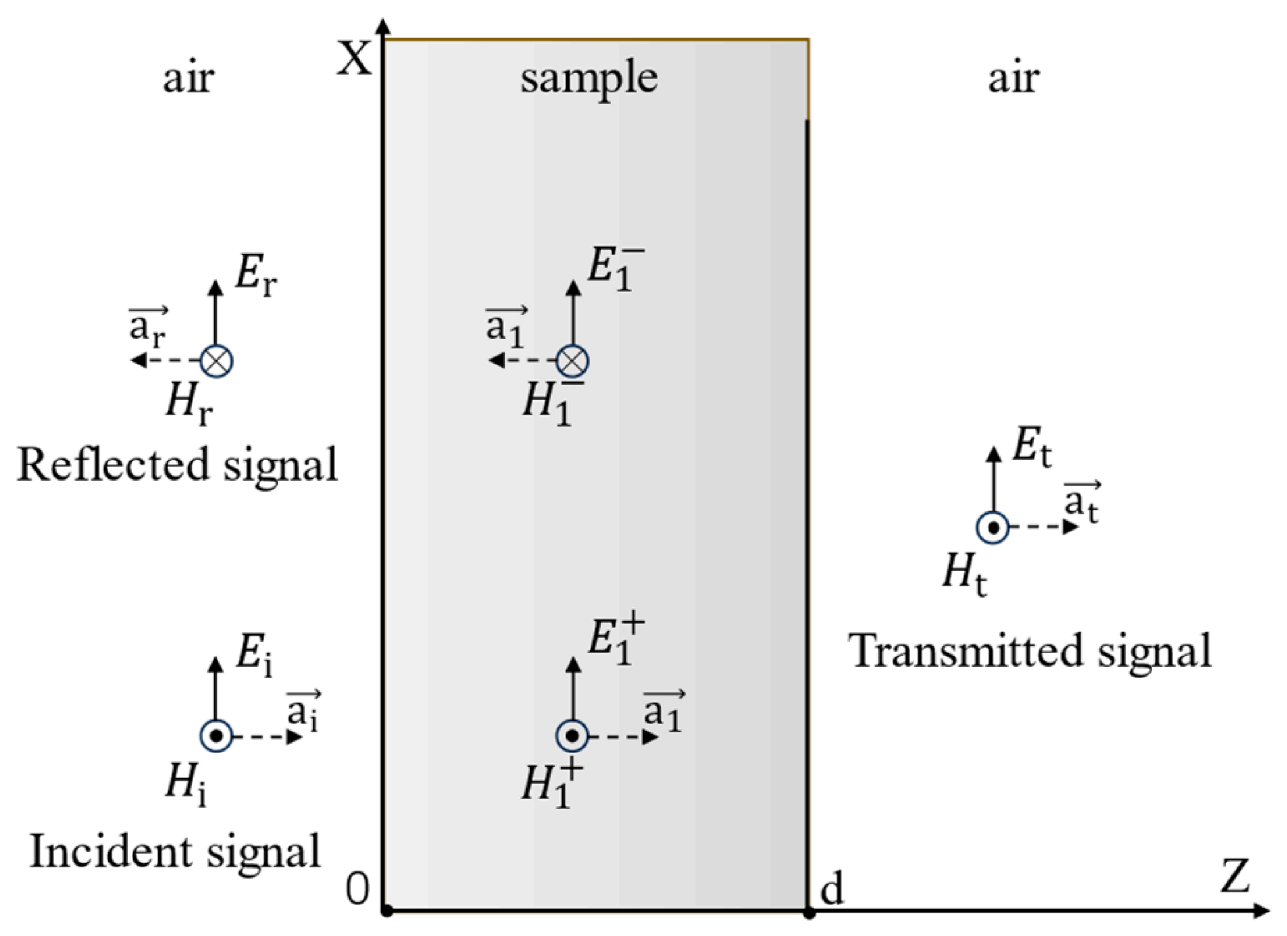
Microwave propagation in a finite-thickness wood specimen.

In the free-space reflection method, the propagation of electromagnetic waves through wood is governed by its effective dielectric properties. Moisture-induced changes in the effective permittivity therefore modify the magnitude and phase of the reflected electromagnetic field. To establish a simplified theoretical description of this interaction, the incident microwave field is approximated as a uniform plane wave normally incident on a planar wood slab. This idealization facilitates the analysis of interface reflections and wave propagation within the material but does not explicitly account for the wavefront curvature, spatially nonuniform illumination, or antenna–specimen coupling associated with a practical horn antenna. A specimen of finite thickness d is considered, as illustrated in Figure 1.

The regions z < 0 and z > d are assumed to be air, with permittivity *ε*_0_ and permeability μ_0_, whereas the region 0 < z < d corresponds to wood, with effective permittivity *ε*_eff_ and permeability *μ*_1_. The tangential electric and magnetic fields satisfy the continuity conditions at the two interfaces. Here, *E_i_* and *H_i_* denote the incident fields; *Eᵣ* and *Hᵣ* denote the reflected fields; *E*_1_^+^ and *H*_1_^+^ denote the forward-propagating fields within the wood; *E*_1_^−^ and *H*_1_^−^ denote the backward-propagating fields; and *E_t_* and *H_t_* denote the transmitted fields.

At z = 0, the boundary conditions are

(3) $$E_{i}+E_{r}=E_{1}^{+}+E_{1}^{-}$$

(4) $$H_{i}-H_{r}=H_{1}^{+}-H_{1}^{-}$$

At z = d, the boundary conditions are

(5) $$(E_{1}^{+}+E_{1}^{-})*e^{\left(-\alpha_{1}-j\beta_{1}d\right)}=E_{t}$$

(6) $$(H_{1}^{+}-H_{1}^{-})*e^{\left(-\alpha_{1}-j\beta_{1}d\right)}=H_{t}$$

(7) $$\alpha_{1}-j\beta_{1}=\omega \sqrt{\mu_{1}\varepsilon_{\mathrm{eff}}}$$

where *ω* = 2*πf* is the angular frequency, and *f* is the operating frequency, and *α*_1_ and *β*_1_ are, respectively, the attenuation and phase constants of the wave propagating in the wood. By simultaneously solving the boundary conditions in Equations (3)–(6) and incorporating the propagation relationship in Equation (7), the plane-wave reflection coefficient of the finite-thickness wood slab can be obtained as follows, consistent with the theoretical formulation reported by [37]:

(8) $$S_{11}=\frac{E_{r}}{E_{i}}=\frac{\frac{\sqrt{\frac{1}{\varepsilon_{\mathrm{eff}}}}-1}{\sqrt{\frac{1}{\varepsilon_{\mathrm{eff}}}}+1}\left(1-e^{-j\frac{4\pi }{\lambda_{0}}\sqrt{\varepsilon_{\mathrm{eff}}}\;d}\right)}{1-e^{-j\frac{4\pi }{\lambda_{0}}\sqrt{\varepsilon_{e\mathrm{ff}}}\;d}{\left(\frac{\sqrt{\frac{1}{\varepsilon_{\mathrm{eff}}}}-1}{\sqrt{\frac{1}{\varepsilon_{\mathrm{eff}}}}+1}\right)}^{2}}$$

where *λ*_0_ is the free-space wavelength and *S*_11_ denotes the idealized plane-wave reflection coefficient of the dielectric slab. Equation (8) describes the dependence of the slab reflection response on its effective permittivity under normal plane-wave incidence. However, this coefficient is not directly equivalent to the one-port reflection coefficient S_11_ measured at the horn antenna feed, which is additionally influenced by the antenna characteristics, spatial field distribution, propagation distance, and antenna–specimen interactions. These effects are considered in the full-wave numerical model described in Section 3 and are incorporated into the experimentally established calibration relationships.

Equation (8) demonstrates that S_11_ depends explicitly on the effective permittivity of the wood slab. Because the effective permittivity varies with moisture content, the measured reflection response can be related to moisture content through the following composite mapping:

*S*_11_ = *G_s_*[*ε_e_*_ff_(*θ*)] (9)

where *ε*_eff_ (*θ*) denotes the moisture-dependent effective permittivity of wood, and G denotes the measurement-system mapping determined by the antenna configuration, electromagnetic-field distribution, antenna-to-specimen distance, and slab geometry. In practice, the combined effects of wood heterogeneity, anisotropy, and the measurement configuration make this relationship difficult to describe accurately with a single closed-form expression; it is therefore estimated empirically from the measured reflection features.

## 3. Simulation

### 3.1. Simulation Model and Parameter Settings

**Figure 2:**
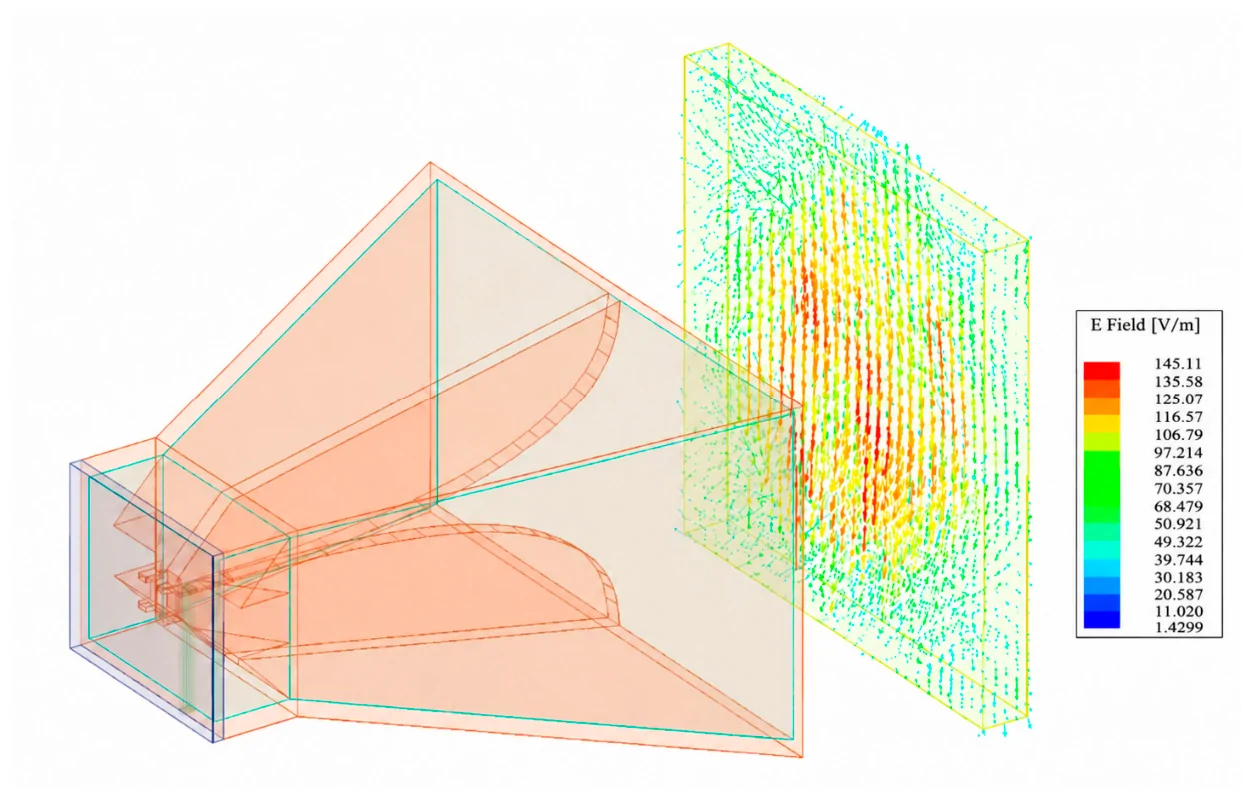
HFSS model of the double-ridged horn antenna and wood specimen.

A three-dimensional full-wave model was developed using Ansys High Frequency Structure Simulator (HFSS) to assess the feasibility of free-space microwave reflection for wood moisture evaluation and to support the selection of key experimental parameters. As shown in Figure 2, the model consisted of a coaxially fed double-ridged horn antenna and a finite-sized wood plate. The electromagnetic-field distribution and the one-port reflection coefficient, *S*_11_, were calculated for each prescribed moisture condition, thereby enabling the moisture-dependent variations in the reflection spectrum to be examined.

The simulations were performed using the Driven Modal solution type with the Network Analysis option in Ansys HFSS. An interpolating frequency sweep was employed over the range of 0.5–6 GHz, with 451 frequency points specified for the sweep.

The double-ridged horn antenna was selected because its gradual transition from the ridged waveguide to free space provides broadband impedance matching and relatively stable radiation characteristics over the investigated frequency range. In the proposed one-port configuration, the same antenna was used to transmit the incident microwave signal and receive the reflected response. This single-antenna arrangement reproduces the single-sided inspection geometry required for wooden components whose rear surfaces are inaccessible, as is frequently encountered in the in-situ examination of wooden cultural heritage.

To isolate the dielectric effect associated with moisture variation, the wood specimen was represented as a homogeneous and isotropic rectangular plate with a moisture-dependent effective complex permittivity. The specimen dimensions were 200 mm × 200 mm × 20 mm. The thickness of 20 mm was selected based on preliminary measurements of the wooden component intended for the in-situ investigation, whose thickness was approximately 20 mm. Accordingly, this thickness was adopted in both the numerical simulations and laboratory experiments to maintain consistency with the target application. The lateral dimensions were selected to cover the principal illuminated region of the antenna and to reduce the influence of edge diffraction on the simulated reflection response. This idealized representation was not intended to reproduce the full anisotropic and heterogeneous dielectric behavior of natural wood; rather, it was used to identify moisture-sensitive spectral features and to guide the subsequent experimental design. The simulated reflection spectra were examined to identify local peaks and valleys exhibiting systematic moisture-dependent variations. The antenna-to-specimen standoff distance was varied from 20 to 200 mm in increments of 20 mm, while all other model parameters were kept constant, to evaluate its influence on the linearity and moisture sensitivity of the selected reflection feature.

Moisture contents ranging from 3% to 30% were considered in the numerical simulations. To represent the dielectric variations associated with different moisture levels, the effective complex permittivity of wood was assigned based on the moisture-dependent dielectric data reported in the literature. In particular, Rönnow et al. [36] summarized the real and imaginary components of the complex permittivity of wood as functions of moisture content, including experimental data at different frequencies and polarization orientations. Referring to these reported relationships, representative dielectric parameters were adopted for the investigated moisture levels, as summarized in Table 1. Both the real and imaginary components increase with moisture content. These parameters were assigned to the homogeneous and isotropic wood model in HFSS to investigate the resulting changes in the reflection response. This simplified representation was intended to capture the principal moisture-dependent dielectric trends rather than the full species-dependent, anisotropic, and frequency-dispersive behavior of natural wood.

### 3.2. Simulated Reflection Response and Definition of Valley Depth

**Figure 3:**
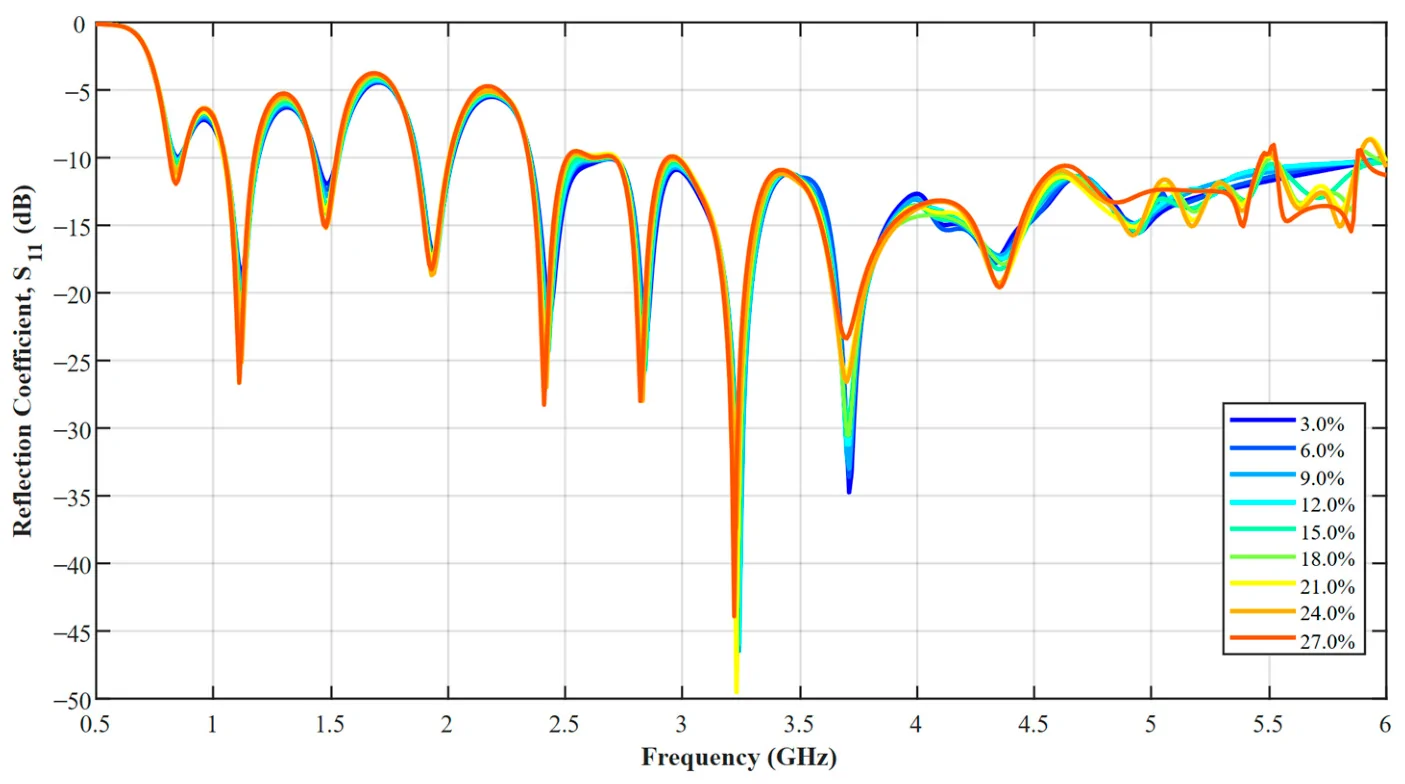
Simulated S_11_ spectra of wood at different moisture contents.

Figure 3 presents the simulated *S*_11_ spectra of the wood specimen at different moisture contents. Systematic moisture-dependent changes are most evident in the local peaks and valleys below approximately 3.5 GHz. Above this frequency, the curves increasingly overlap and cross one another, indicating that the reflection magnitude becomes less monotonic with moisture content and is therefore less suitable for direct quantitative characterization. Within the low-frequency region, the second valley near 1.10 GHz and the adjacent peak near 1.29 GHz exhibit a particularly clear opposite response. As moisture content increases, the valley becomes deeper, corresponding to a more negative *S*_11_ value, whereas the adjacent peak becomes higher, corresponding to a less negative *S*_11_ value. This opposite variation indicates that moisture changes the local spectral morphology rather than merely shifting the entire reflection curve in one direction.

**Figure 4:**
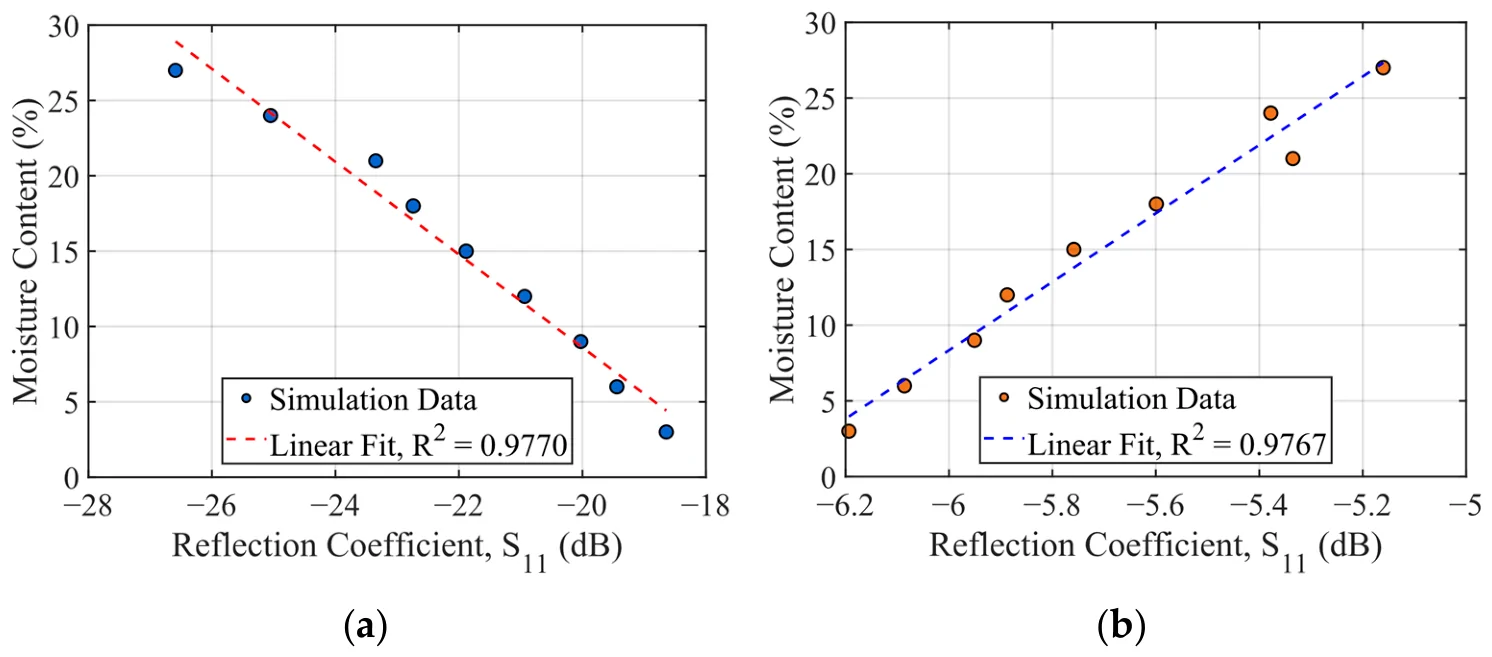
Linear relationships between moisture content and (**a**) the selected valley magnitude and (**b**) the adjacent peak magnitude.

To quantify the two trends identified in Figure 3, the S_11_ magnitudes at the valley near 1.10 GHz and at the adjacent peak near 1.29 GHz were extracted from every simulated moisture-content curve and fitted separately. Thus, the data in Figure 4 were obtained directly from the spectra shown in Figure 3 rather than from an additional simulation. Both individual features exhibit an approximately linear relationship with moisture content, but their fitted slopes have opposite signs.

Because the two neighboring spectral features vary in opposite directions, their amplitude difference was used to represent the local valley morphology. The valley-depth feature, D, was defined as:

D = Γ_peak_ − Γ_valley_ (10)

where Γ_peak_ and Γ_valley_ denote the *S*_11_ magnitudes, expressed in decibels, at the adjacent peak near 1.29 GHz and the valley near 1.10 GHz, respectively. Taking the difference between neighboring points enhances their moisture-dependent contrast and can partially suppress disturbances that affect the local reflected level in a similar manner, such as overall amplitude drift and common-mode fluctuations.

To determine whether this differential representation was more effective than an individual spectral point, the valley magnitude, peak magnitude, and valley-depth feature for the representative 1.10–1.29 GHz peak–valley pair were each related to the reference moisture content using a univariate linear calibration model:

MC = *m*_1_*X* + *m*_2_ (11)

where MC denotes the wood moisture content; *X* is the reflection feature and represents Γ_peak_, Γ_valley_, or D; and m_1_ and m_2_ are the slope and intercept of the fitted model, respectively.

The fitting results are summarized in Table 2. Linearity was evaluated using the coefficient of determination, *R*^2^. Sensitivity was defined as the change in the reflection feature per unit change in moisture content. Because moisture content was regressed on the reflection feature, the sensitivity *S* was calculated as 1/|*m*_1_| and expressed in dB/%.

The valley-depth feature provided the highest *R*^2^ and sensitivity among the three evaluated representations. Compared with a single peak or valley magnitude, the differential feature amplifies the moisture-induced local contrast while reducing the influence of disturbances shared by the neighboring spectral points. Valley depth was therefore selected as the basic reflection feature. At this stage, the 1.10-GHz valley and 1.29-GHz peak served as a representative pair for defining and validating the feature form; the optimal valley frequency was subsequently determined by applying the same definition to all candidate valleys in Section 3.3.

### 3.3. Optimization of Feature Frequency and Standoff Distance

**Figure 5:**
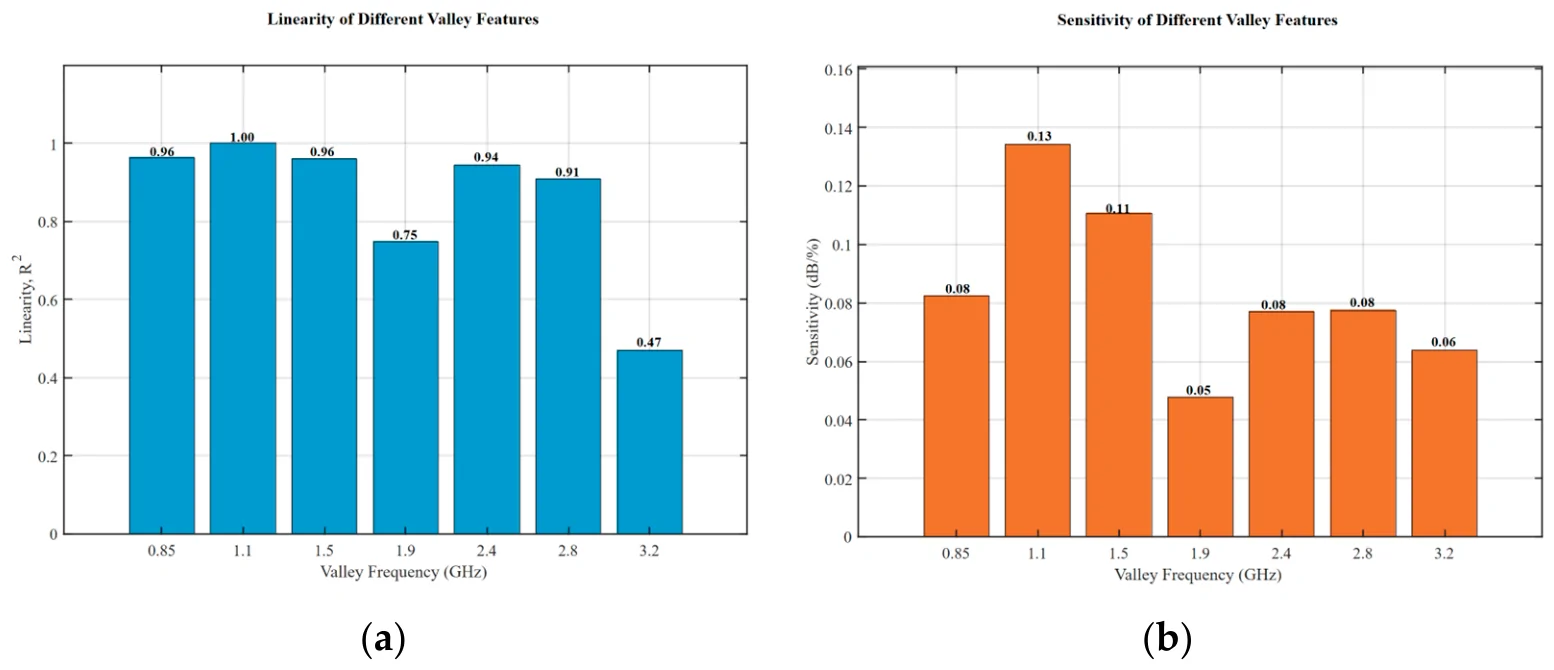
Comparison of valley-depth features at different frequencies: (**a**) coefficient of determination and (**b**) sensitivity.

After valley depth had been established as the preferred feature form, the same extraction and fitting procedure was applied to seven valleys located at approximately 0.85, 1.10, 1.50, 1.90, 2.40, 2.80, and 3.20 GHz. For each candidate valley, its depth relative to the adjacent peak was calculated at every moisture content and then fitted using the linear model in Equation (11). As shown in Figure 5, the selected valley frequency strongly affected both *R*^2^ and sensitivity. The second valley near 1.10 GHz provided the best combined performance and was therefore selected as the operating feature for the subsequent distance analysis and experiments. This comparison also confirms that the representative peak–valley pair used in Section 3.2 corresponds to the most suitable candidate among the seven evaluated valleys.

**Figure 6:**
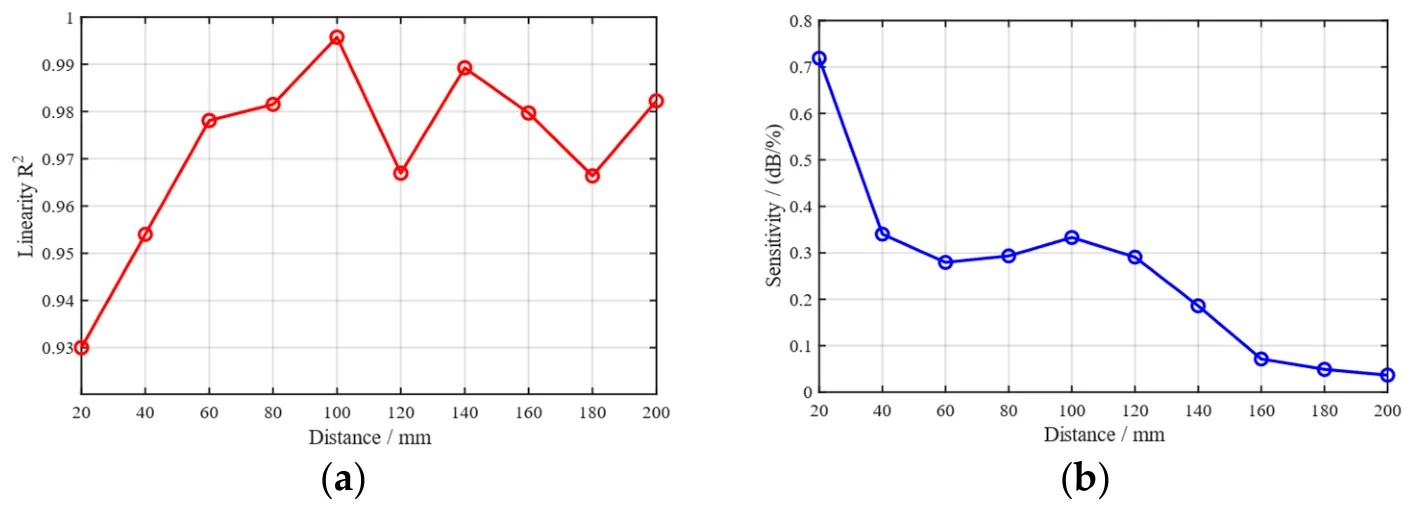
Effect of antenna-to-sample distance on (**a**) coefficient of determination and (**b**) sensitivity.

With the valley frequency fixed near 1.10 GHz, the antenna-to-specimen standoff distance was then varied from 20 to 200 mm. Short standoff distances produced large feature changes but lower linearity and greater susceptibility to the complex antenna–specimen coupling in the near field. As the distance increased, the sensitivity generally decreased because the reflected response became weaker. Figure 6 shows that a standoff distance of 100 mm provided a favorable compromise, combining high *R*^2^ with appreciable sensitivity. This distance was therefore selected for the laboratory measurements. Accordingly, the subsequent experiments used the valley-depth feature formed by the valley near 1.10 GHz and its adjacent peak near 1.29 GHz.

**Figure 7:**
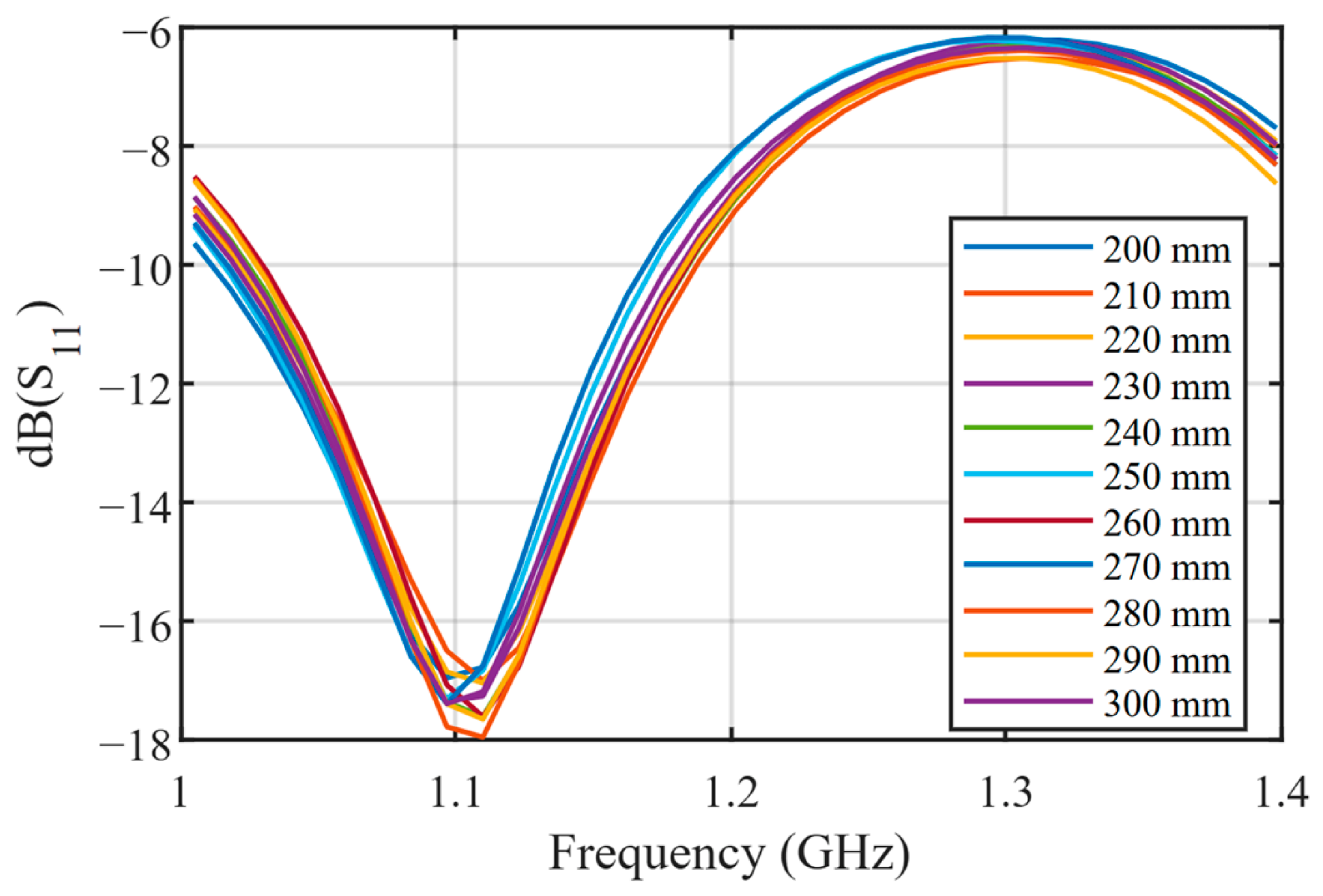
Influence of specimen lateral dimensions on the valley-depth feature.

To investigate the influence of specimen lateral dimensions on the reflection response, additional simulations were conducted using square wood specimens with different side lengths while maintaining the other simulation parameters unchanged. As shown in Figure 7, variations in specimen size result in slight changes in the reflection spectra and the corresponding valley-depth feature. However, the overall spectral characteristics remain similar, and the positions of the selected valley and adjacent peak exhibit no substantial shifts. These results suggest that, although specimen lateral dimensions affect the reflection response, their influence on the selected spectral features is relatively limited within the investigated size range.

## 4. Materials and Experiments

### 4.1. Free-Space Reflection Measurement System

**Figure 8:**
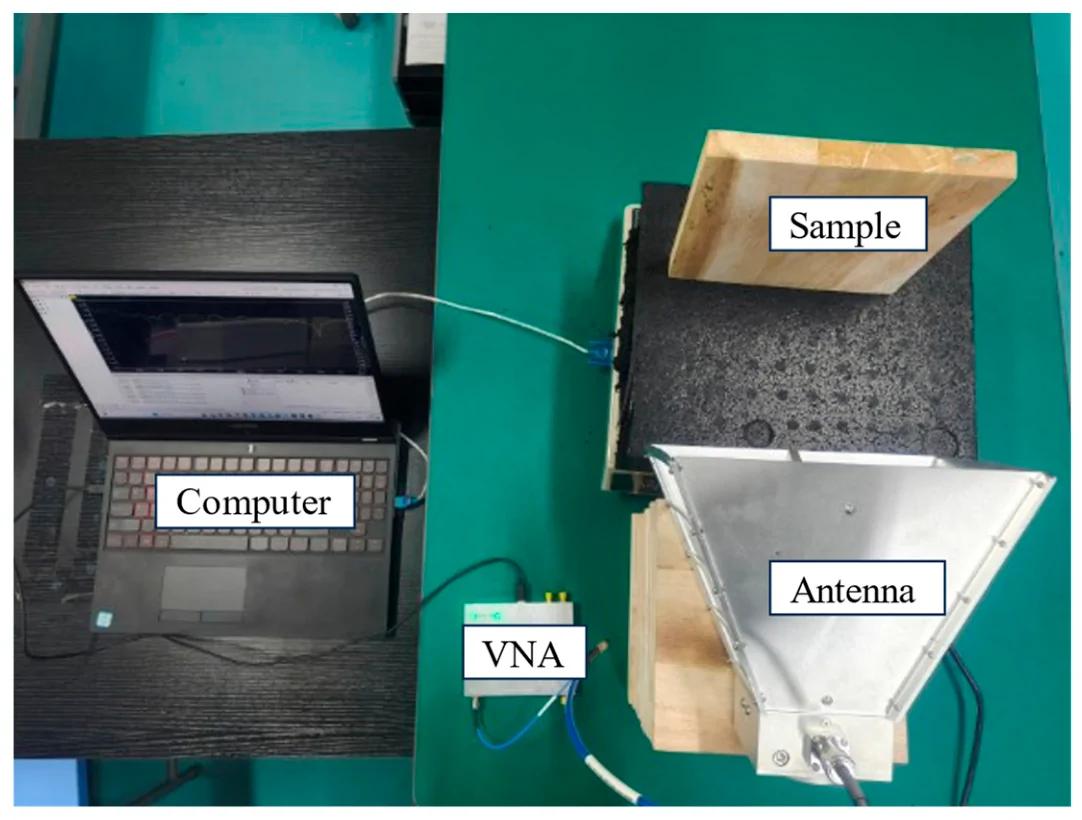
Schematic of the free-space reflection measurement system.

A free-space microwave reflection system was constructed to experimentally evaluate the dielectric sensitivity of the selected valley-depth feature and its applicability to wood moisture assessment. As shown in Figure 8, the system consisted of a portable vector network analyzer (VNA), a coaxially fed double-ridged horn antenna, and a computer for instrument control and data acquisition. The double-ridged horn antenna was connected to the VNA through a coaxial cable and was used for both microwave transmission and reception. The incident field was directed toward the front surface of the specimen, while the reflected signal was received by the same antenna. This one-port configuration requires access to only one side of the inspected object and therefore avoids the rear-surface accessibility required by conventional free-space transmission measurements. Such a configuration is particularly suitable for the in-situ examination of fixed or partially inaccessible wooden heritage components. During measurement, the center of the specimen was aligned with the antenna boresight. Based on the numerical analysis presented in Section 3, the aperture-to-specimen standoff distance was fixed at 100 mm for all laboratory measurements. The specimen position and antenna orientation were kept unchanged during each series of measurements to minimize variations associated with distance, lateral displacement, and incidence angle. The same instrumental settings and measurement geometry were used in both the reference-material and wood-moisture experiments to ensure comparability between the different stages of experimental validation. The portable VNA supported a frequency range of 1 MHz to 6 GHz. In this study, the reflection coefficient *S*_11_ was measured over 0.5–6 GHz with 1001 frequency points per sweep. Before data acquisition, a one-port short–open–load (SOL) calibration was performed, with the calibration reference plane established at the antenna feed connection.

### 4.2. Reference Dielectric Materials Measurement

#### 4.2.1. Reference Dielectric Materials

Polytetrafluoroethylene (PTFE), polypropylene (PP), polyoxymethylene (POM), and flame-retardant grade 4 (FR4) glass-fiber-reinforced epoxy laminate were used as reference dielectrics, with relative permittivities of 2.08, 2.26, 2.73, and 4.40, respectively. Their dielectric properties are comparatively stable over the measurement band, and the selected values span the principal effective-permittivity range of wood below the fiber saturation point. Each reference plate measured 200 mm × 200 mm × 20 mm, matching the simulated specimen.

#### 4.2.2. Measurement and Evaluation Methods

For the reference-material experiment, PTFE, PP, POM, and FR4 were placed sequentially in front of the antenna. The relevant geometric position settings are the same as those in 4.1. After the response stabilized, *S*_11_ was acquired over 0.5–6 GHz. The depth of the second valley near 1.1 GHz relative to its adjacent peak was extracted and correlated with relative permittivity. A univariate linear regression model was established using valley depth D as the input and relative permittivity as the output:

(12) $$\varepsilon^{\prime }=a_{1}D+a_{2}$$

where *ε*′ denotes the real part of the relative permittivity of the reference material, D is the extracted valley-depth feature, and a_1_ and a_2_ are the regression coefficients. This analysis was conducted to evaluate the sensitivity of the selected reflection feature to dielectric-property variations.

#### 4.2.3. Results of Dielectric Materials Experiment

**Figure 9:**
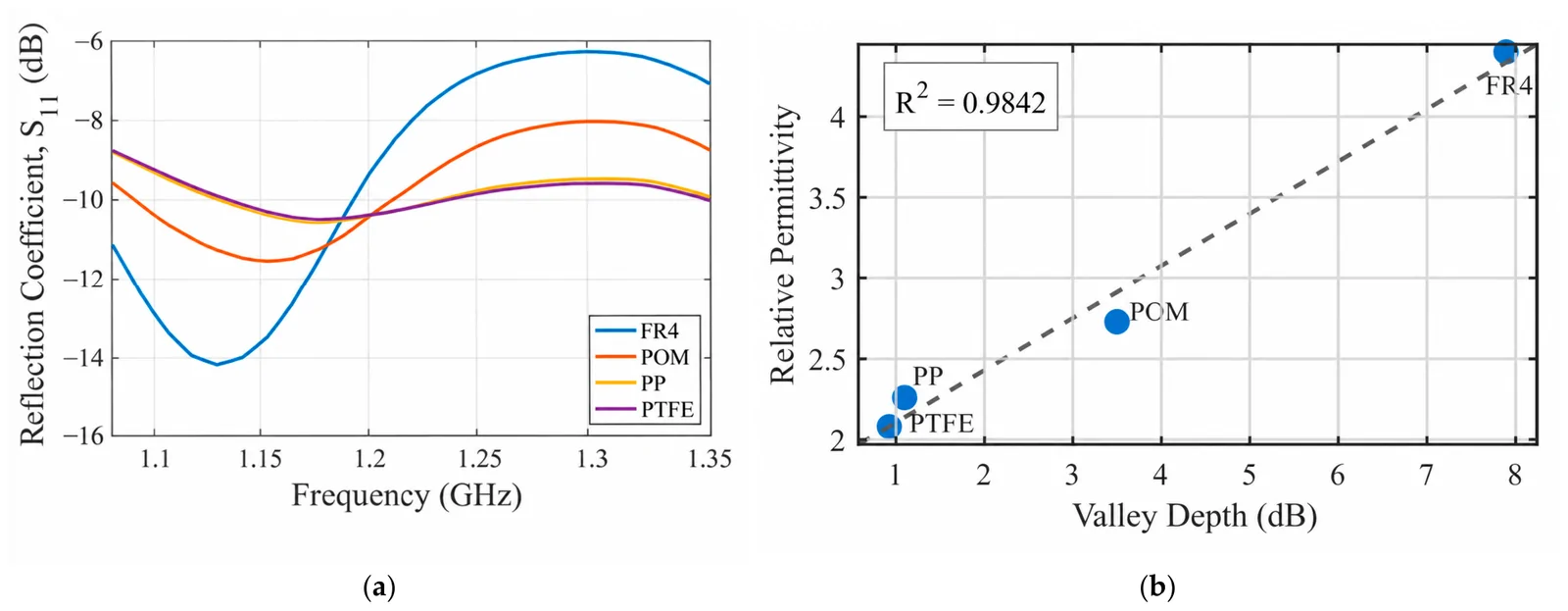
Reference dielectric measurements: (**a**) S_11_ spectra and (**b**) linear relationship between valley depth and relative permittivity.

The measured S_11_ spectra of the reference materials are shown in Figure 9a. Systematic variations with relative permittivity were evident over 0.6–2.0 GHz, whereas stronger curve crossings occurred at higher frequencies. The extracted depth of the second valley near 1.1 GHz increased with relative permittivity and yielded *R*^2^ = 0.98 (Figure 9b). This result confirms that the experimental system can convert dielectric-property changes into a stable measurable reflection feature.

### 4.3. Wood Moisture Measurement

#### 4.3.1. Wood Specimens and Moisture Conditioning

Pine and paulownia were selected as representative wood species. Five specimens of each species were used for calibration, and an additional three specimens of each species were prepared for independent validation. All specimens measured 200 mm × 200 mm × 20 mm. The reference moisture content was calculated from the oven-dry mass according to the standardized oven-dry procedure:

(13) $$\mathrm{MC}=\frac{m_{1}-m_{0}}{m_{0}}\times 100\%$$

where m is the specimen mass at measurement and m_0_ is the constant oven-dry mass. Specimens were first dried to constant mass to determine m_0_ and were then conditioned by water uptake to a moisture level near the fiber saturation point. The investigated range was limited to below 30%, covering the principal moisture interval below fiber saturation. Specifically, to prepare specimens with different moisture contents, all wood specimens were initially oven-dried in accordance with GB/T 1931-2009. During drying, the specimens were weighed at proper intervals until a constant mass was obtained, which was recorded as the oven-dry mass (m_0_). Subsequently, all specimens were immersed in water until their moisture content reached approximately 30%. Each specimen was then wrapped in black polyethylene (PE) film and left undisturbed for at least 2–3 days to minimize moisture evaporation and promote a more uniform moisture distribution within the wood. Finally, the sample with a high humidity level was obtained.

The experimental system for measuring wood moisture and the data evaluation method are consistent with those mentioned in Section 4.1 and Section 4.2.2. It should be noted that wood measurements were performed in two orientations. The x-direction denotes an electric field parallel to the grain, whereas the y-direction denotes an electric field perpendicular to the grain. Starting from a high-moisture state, the specimens were dried stepwise in an oven at 90 °C. After each drying interval, they were equilibrated at room temperature for 30 min, weighed, and measured immediately. Three repeated S_11_ sweeps were recorded for each moisture state and orientation and averaged. The procedure was repeated until a set of moisture states below 30% had been obtained. Independent validation specimens followed the same conditioning and measurement protocol.

When species and grain orientation were known, separate models were established for pine-x, pine-y, paulownia-x, and paulownia-y. Independent-sample prediction performance was evaluated using the root mean square error (RMSE) and mean absolute error (MAE):

(14) $$\mathrm{RMSE}=\sqrt{\frac{1}{n}\overset{n}{\underset{i=1}{\sum }}{\left({\overset{\wedge }{\mathrm{MC}}}_{i}-{\mathrm{MC}}_{i}\right)}^{2}}$$

(15) $$\mathrm{MAE}=\frac{1}{n}\overset{n}{\underset{i=1}{\sum }}\left|{\overset{\wedge }{\mathrm{MC}}}_{i}-{\mathrm{MC}}_{i}\right|$$

#### 4.3.2. Results of Wood Moisture Experiment

**Figure 10:**
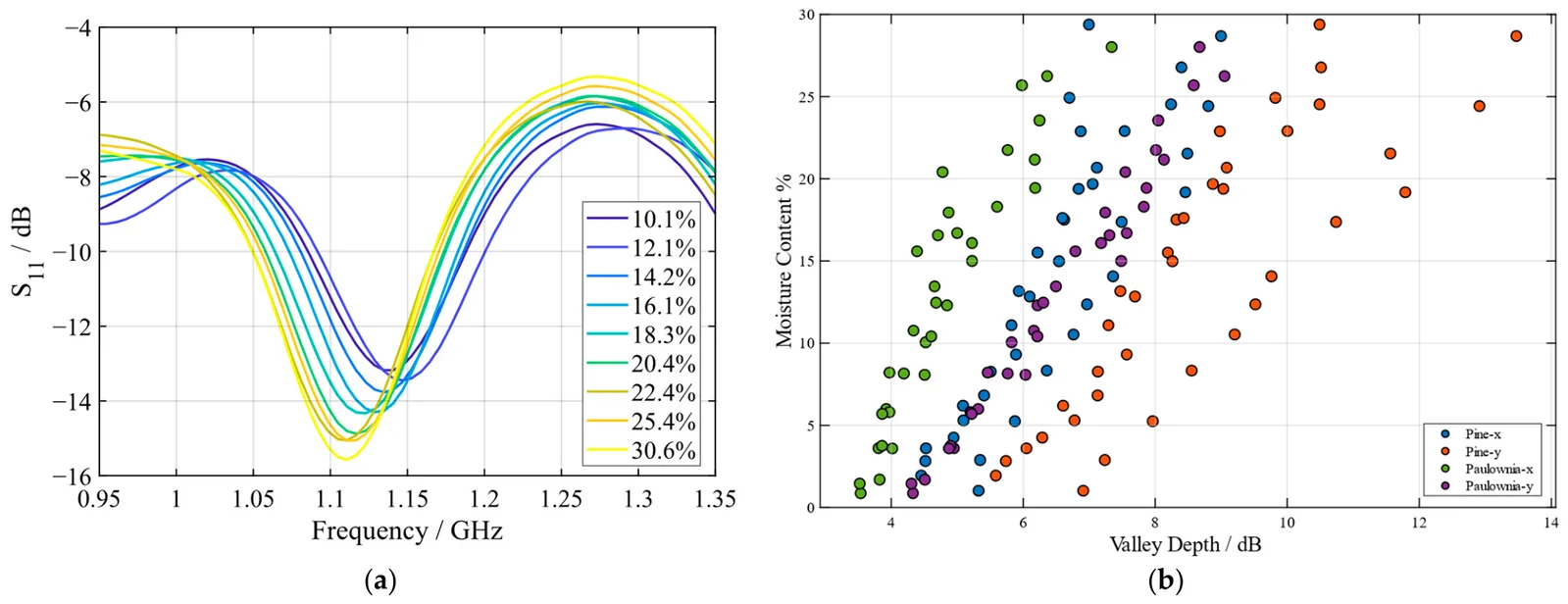
Wood measurements: (**a**) representative S_11_ spectra at different moisture contents and (**b**) valley depth versus moisture content for the four species-orientation groups.

Representative S_11_ spectra of a wood specimen at different moisture states are shown in Figure 10a. Several low-frequency peaks and valleys changed systematically with moisture content, while the high-frequency response exhibited more pronounced fluctuations and curve crossings, consistent with the simulations. Valley-depth slopes and scatter differed (Figure 10b), demonstrating that both species and grain orientation influence the free-space reflection response.

To evaluate the repeatability of the free-space reflection measurement system, the pine-x, pine-y, paulownia-x, and paulownia-y groups were analyzed separately. For each category, the mean valley depth was first calculated from three replicate measurements acquired for each specimen at each moisture state. The residual of each individual measurement relative to its corresponding mean was then determined. Finally, all residuals from all specimens and moisture states within the same category were pooled, and their standard deviation was calculated as the repeatability metric:

(16) $$\sigma_{\mathrm{rep}}=\sqrt{\frac{\sum_{j=1}^{5}\sum_{g=1}^{G}\sum_{i=1}^{n}{\left(D_{j,g,i}-{\bar{D}}_{j,g}\right)}^{2}}{\sum_{j=1}^{5}\sum_{g=1}^{G}\left(n-1\right)}}$$

where *G* denotes the number of valid measurement groups for each specimen, j is the specimen index, and n is the number of replicate measurements in each group (n = 3 in this study). *D_j,g,i_* denotes the valley depth obtained from the ith replicate measurement in the *g*th measurement group of the *j*th specimen, whereas *D_j,g_* represents the mean valley depth of the three replicate measurements for the corresponding specimen and group.

The repeatability standard deviations for pine-x, pine-y, paulownia-x, and paulownia-y were 0.011, 0.013, 0.008, and 0.011 dB, respectively. The repeatability standard deviation therefore accounted for only about 0.2–0.3% of the full feature variation, indicating good short-term measurement stability.

The group-specific calibration results are presented in Table 3. When species and orientation were known, all four groups exhibited a measurable linear relationship, with paulownia-y giving the highest *R*^2^ (0.98) and pine-y the lowest (0.77). The stronger linearity observed for paulownia may be associated with its relatively open structure and more uniform moisture redistribution. In contrast, resin canals, density variation, and local heterogeneity in pine may introduce additional scattering and dielectric variability.

The influence of grain orientation was not limited to sensitivity. For example, pine-y exhibited a higher sensitivity (0.24 dB/%) than pine-x, but a lower *R*^2^. Thus, a larger feature change does not necessarily imply a more stable quantitative relationship. Changing the electric-field direction alters polarization at the cell walls, lumina, and moisture-containing interfaces, leading to orientation-dependent dielectric behavior.

Independent-sample validation results are listed in Table 4. Under known species and orientation, the paulownia-y model achieved the lowest errors, with RMSE = 1.27% and MAE = 0.99%, whereas the pine-y model produced the largest group-specific errors. The valley-depth feature therefore captures moisture changes effectively within a defined material group, but a single linear model cannot fully separate moisture effects from species and anisotropy when the category is unknown.

The reflection configuration requires access to only one side of the object and offers non-contact operation, simple hardware, and strong potential for in situ use. Nevertheless, the present experiments used regular, flat pine and paulownia plates under controlled conditions. Actual wooden heritage components may exhibit irregular surfaces, internal damage, ageing, coatings, moisture gradients, and environmental clutter. Future work may exploit the multi-spectral information and local curve morphology, together with nonlinear or AI-based models, to improve generalization across unknown species, orientations, and field conditions.

### 4.4. In-Situ Testing

**Figure 11:**
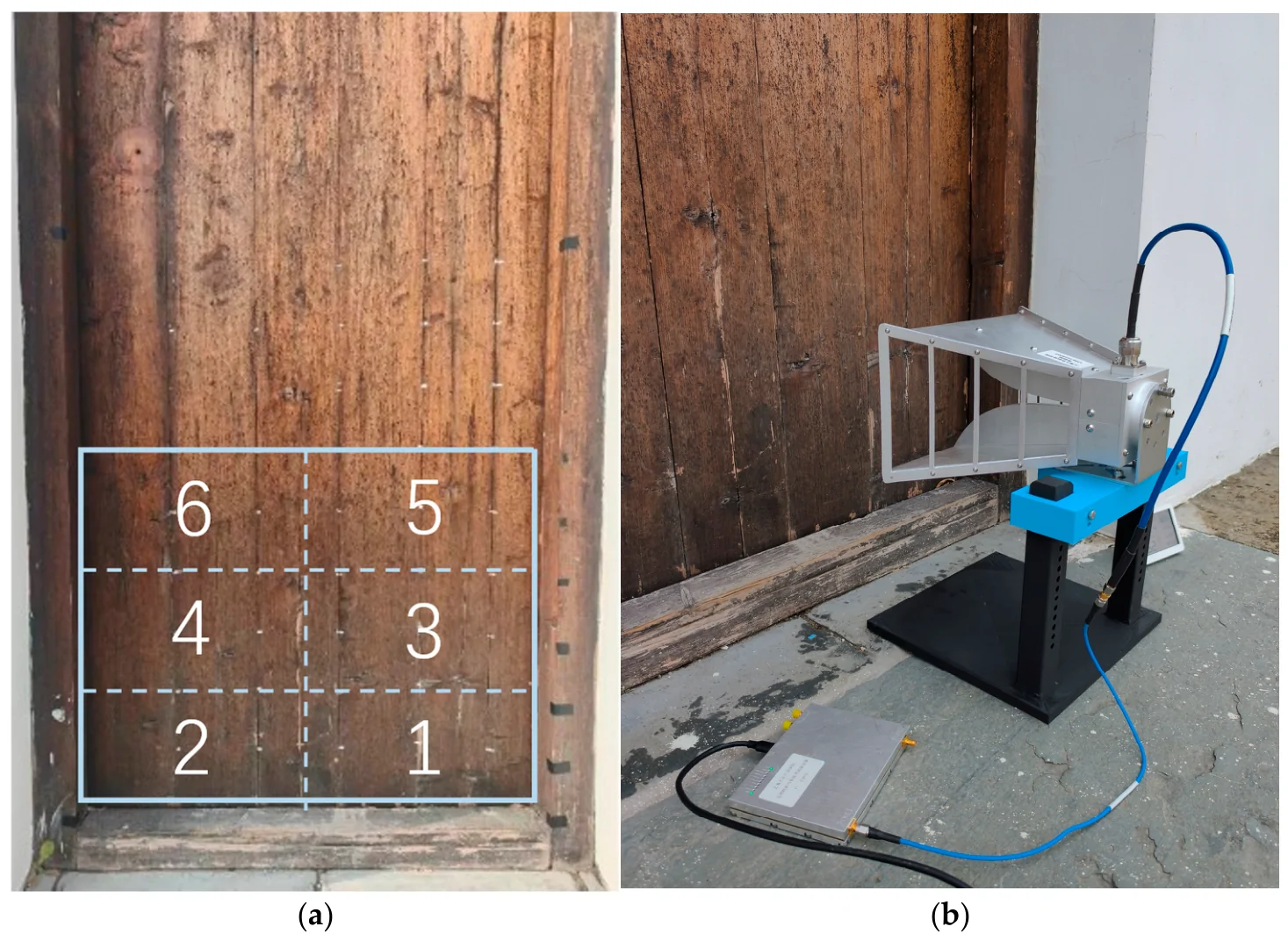
In-situ measurement of the outdoor wooden door: (**a**) selected inspection region divided into six measurement cells. (The numbers represent the identification codes of the measured areas); (**b**) field arrangement of the double-ridged horn antenna and portable VNA.

To further evaluate the practical applicability of the investigated free-space reflection approach, an in-situ test was conducted on an outdoor wooden door of a historical building in Shanghai. As shown in Figure 11, a relatively flat region on the door surface was selected as the inspection area and divided into six rectangular measurement cells arranged in two columns and three rows. The center of each cell was used as the measurement point to ensure spatial consistency among repeated measurements. During the test, the double-ridged horn antenna was positioned approximately normal to the door surface, with the same optimized standoff distance used in the laboratory measurements.

At each measurement point, the reflection coefficient *S*_11_ was acquired over the same frequency range as that used in the laboratory tests. The valley-depth feature near 1.1 GHz was then extracted from each *S*_11_ curve. Because destructive sampling was not permitted for the tested door, the oven-dry moisture content could not be obtained as ground truth. The calibration model obtained from the lab test was used for the retrieval of the moisture.

**Figure 12:**
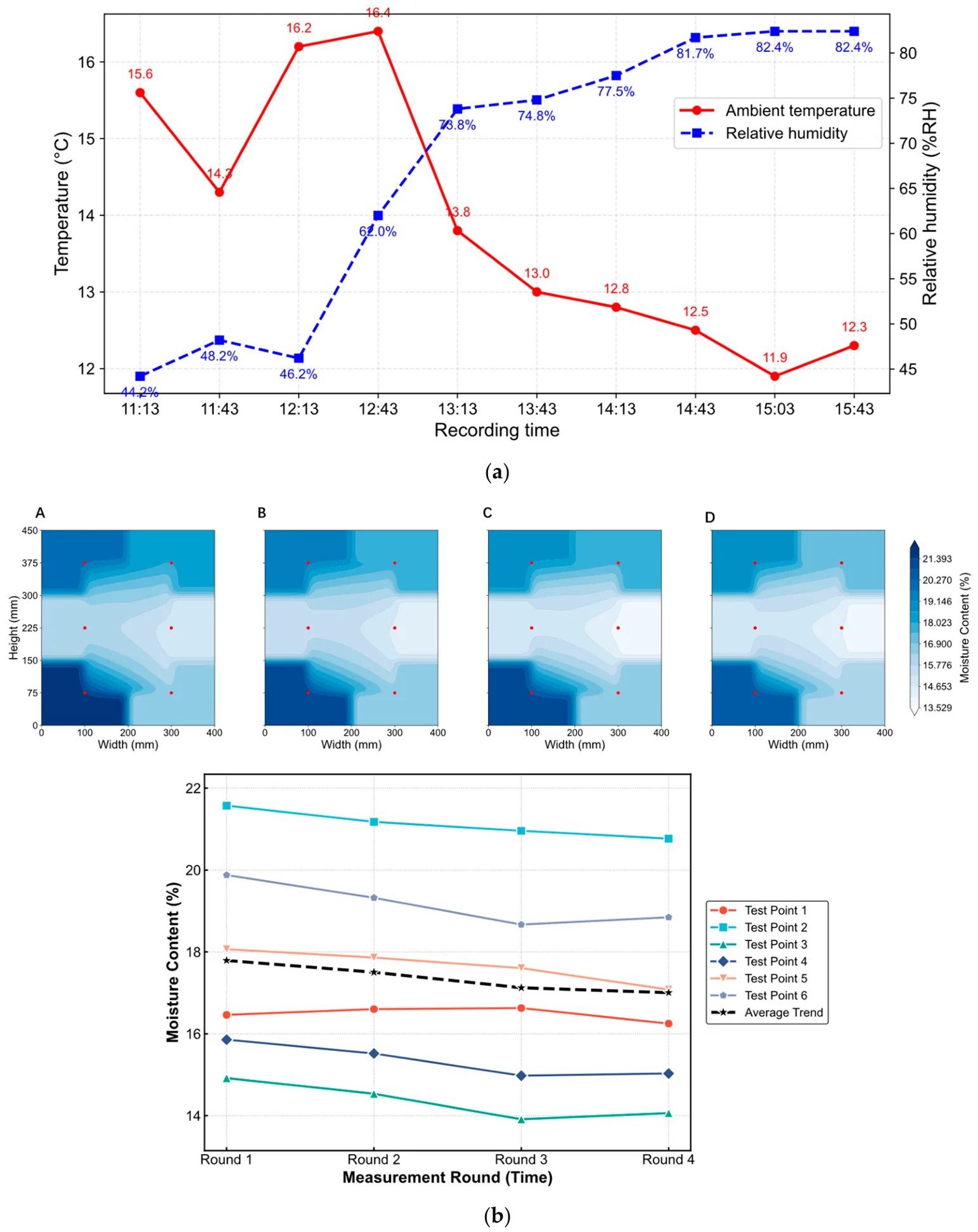
Environmental conditions and estimated moisture-content variations during the in-situ measurement of the outdoor wooden door: (**a**) variations in ambient temperature and relative humidity over the measurement period; (**b**) estimated moisture contents at six measurement points over four measurement rounds, together with their average trend. (A–D represent different detection periods.)

The ambient temperature and relative humidity during the in-situ measurement are shown in Figure 12. During the measurement period, the temperature decreased, whereas the relative humidity increased. This variation indicates that the in-situ test was conducted under non-stationary environmental conditions rather than under a controlled laboratory environment. However, the estimated moisture content of the wooden door exhibited an overall decreasing trend, as shown in Figure 12. This apparent inconsistency does not contradict the hygroscopic behavior of wood, because the moisture state of a wooden component does not respond instantaneously to short-term changes in ambient humidity. Instead, it is affected by the previous environmental exposure, internal moisture diffusion, surface evaporation, coating condition, and local material heterogeneity. Therefore, the measured decreasing trend more likely reflects the existing moisture redistribution and continuing drying state of the door during the test period, rather than a direct response to the instantaneous increase in ambient relative humidity.

The estimated moisture-content maps further show that the moisture distribution over the selected door region was spatially nonuniform. Some local areas exhibited relatively higher estimated moisture content, whereas others remained at lower levels. Such spatial heterogeneity is reasonable for an actual wooden component, where local differences in ageing, surface condition, grain direction, coating, and internal structure may lead to nonuniform moisture accumulation. Compared with regular laboratory specimens, the outdoor door introduced additional uncertainties, including unknown wood species, unknown grain orientation, surface roughness, possible coating layers, and environmental disturbances. Therefore, the in-situ results should be interpreted mainly as relative moisture-distribution indicators rather than strictly calibrated absolute moisture contents.

The in-situ experiment demonstrates that the proposed free-space reflection method can be applied to a real wooden component in a single-sided and non-contact manner. The method can rapidly provide spatial moisture-distribution information under field conditions.

## 5. Discussion

### 5.1. Effectiveness of the Valley-Depth Feature and Influence of Wood Variability

The simulation, reference-dielectric measurements, and wood experiments collectively demonstrate that the valley-depth feature near 1.1 GHz provides a stable indicator of moisture-induced dielectric variation. In the finite-thickness configuration, the measured reflection response results from the coherent superposition of the front-surface reflection and the components propagating within the specimen. Changes in moisture content alter the effective permittivity, interface mismatch, propagation phase, and dielectric attenuation, thereby modifying the local spectral morphology rather than producing only a uniform amplitude shift. The opposite variations in the selected valley and its adjacent peak therefore provide a physical basis for defining their amplitude difference as the valley-depth feature. Because the two points are extracted from neighboring regions of the same spectrum, this differential feature can partially suppress common disturbances, such as overall amplitude drift, cable variation, and minor calibration fluctuations. Its effectiveness is supported by the higher simulated linearity and sensitivity compared with the individual peak or valley magnitudes, the strong relationship with relative permittivity in the reference-material experiment, and the small repeatability standard deviations of 0.008–0.013 dB in the wood measurements. However, the wood results also show that valley depth is governed not only by moisture content but also by species-dependent and orientation-dependent dielectric properties. Variations in density, porosity, anatomical structure, extractives, grain direction, and internal moisture distribution produced different calibration slopes, intercepts, and prediction errors among the four species-orientation groups. These findings indicate that the selected feature is reliable within a defined calibration domain, but its quantitative transferability decreases when wood species and grain orientation are unknown. This distinction is particularly important for interpreting the subsequent in-situ experiment, in which the inspected component was measured under uncontrolled material and environmental conditions.

### 5.2. Field Applicability, Limitations, and Future Trends

The in-situ experiment represents a practical extension of the simulation and controlled laboratory measurements. The numerical analysis identified the moisture-sensitive valley-depth feature near 1.1 GHz and optimized the antenna-to-specimen standoff distance to balance linearity and sensitivity. The reference-dielectric experiment subsequently confirmed that variations in material permittivity could be reflected by systematic changes in this feature, while the wood experiments demonstrated its relationship with moisture content under controlled species, grain-orientation, thickness, and measurement conditions. Based on these results, the same one-port reflection configuration, standoff distance, frequency range, and feature-extraction procedure were applied to the outdoor wooden door. However, because the material properties and reference moisture contents of the door were unknown, its purpose was primarily to examine whether the proposed system could be deployed under practical conditions and whether the selected reflection feature could provide meaningful spatial and temporal contrast. The principal practical advantage of the proposed configuration is that microwave transmission and reception are performed from the same accessible side of the inspected component. This geometry is more suitable than through-transmission arrangements for doors, wall-integrated members, panels, and other wooden heritage components whose rear surfaces are inaccessible. It also avoids electrode insertion, destructive sampling, and sustained probe contact. The outdoor-door experiment showed that the portable VNA and horn antenna could be operated in a field environment and that repeated measurements at selected positions could be converted into spatial moisture-distribution maps. Although the number of measurement points was limited, the resulting maps exhibited clear spatial differences within the inspected region. This indicates that the method has potential for comparative field screening, such as identifying relatively moisture-sensitive areas, monitoring changes at fixed locations, and prioritizing regions that require more detailed conservation assessment. Specimen geometry is an important factor affecting the free-space microwave reflection response. In the present study, a thickness of 20 mm was selected based on preliminary measurements of the intended inspection object and was consistently adopted in the simulations and laboratory experiments. Additional simulations were also conducted to investigate the influence of specimen lateral dimensions. The results indicate that variations in lateral dimensions have a relatively limited influence on the selected spectral features, whereas specimen thickness may have a more pronounced effect on the reflection response. Therefore, the established calibration models are specific to the investigated specimen geometry and measurement configuration, and their applicability to wooden components with different dimensions requires further validation and, where necessary, recalibration.

The current findings also indicate several directions for further development. The laboratory study was conducted using pine and paulownia specimens with a single nominal thickness and regular flat surfaces, and the quantitative evaluation was mainly restricted to moisture contents below 30%. In addition, the reported repeatability describes short-term repeated sweeps under unchanged positioning conditions and does not yet include the effects of specimen repositioning, long-term instrument drift, temperature variation, or recalibration. A single measurement cannot directly distinguish surface moisture from deeper moisture or resolve a through-thickness moisture gradient. Therefore, future studies should focus on improving model transferability while retaining the simplicity of the present single-sided configuration. A larger specimen database covering additional species, densities, thicknesses, ageing states, coatings, surface geometries, and controlled moisture gradients would help clarify the influence of material and structural variability. From the modeling perspective, the valley-depth feature can be supplemented with information from the broader low-frequency spectrum, including multiple peaks, valleys, frequency shifts, and local curve morphology. Physically informed multivariate, classification-regression, or nonlinear models may then be used to first account for material category or orientation and subsequently estimate moisture content. These developments could gradually extend the proposed method from relative field screening toward more robust quantitative moisture assessment under unknown and heterogeneous heritage conditions.

Another potential direction is the integration of microwave reflection measurements with microwave thermography, which combines microwave-induced heating with infrared imaging to visualize spatially varying thermal responses and has attracted attention in nondestructive testing (NDT) and structural health monitoring (SHM) [38]. Recent studies have demonstrated the effectiveness of active microwave thermography in detecting interfacial debonding in CFRP–timber structures and extended its application to scanning inspection of FRP–timber joints [39,40]. Although these investigations focus on structural defects rather than quantitative moisture assessment, they suggest the potential of combining electromagnetic and thermal responses for more comprehensive evaluation of wooden components. Future research could explore the complementary use of reflection features and thermal imaging for spatial moisture assessment and condition monitoring. However, microwave-induced heating may pose risks to temperature-sensitive wooden heritage objects. Therefore, excitation power, heating duration, and temperature rise must be carefully controlled, potentially through low-energy pulsed or modulated excitation strategies [41].

## 6. Conclusions

This study investigated the application of an established single-sided free-space microwave reflection technique to nondestructive wood moisture assessment, with particular emphasis on the evaluation of a valley-depth feature for single-sided measurements. A double-ridged horn antenna was used to acquire the one-port reflection response, and a valley-depth feature was defined from the magnitude difference between a local valley and its adjacent peak. HFSS simulation identified the valley near approximately 1.1 GHz as a suitable moisture-sensitive feature and indicated that a 100 mm standoff distance provided a favorable balance between linearity and sensitivity. Reference-dielectric measurements verified the relationship between valley depth and dielectric variation. For pine and paulownia specimens below 30% moisture content, the repeatability standard deviation was 0.008–0.013 dB. The best independent-sample result was obtained for paulownia-y, with *R*^2^ = 0.98, RMSE = 1.27%, and MAE = 0.99%. The results demonstrate the feasibility of rapid, single-sided, and non-contact moisture assessment using free-space reflection features. The outdoor-door test further showed that the method can provide spatial moisture-distribution information under field conditions. At present, species- and orientation-specific calibration is preferable for quantitative prediction, while measurements under unknown field conditions are more appropriately used for relative screening and temporal or spatial comparison. The study establishes a basis for the further development of reflection measurements for the in-situ inspection and preventive conservation of wooden cultural heritage.

## Figures and Tables

**Table 1:** Dielectric parameters assigned to the wood specimens at different moisture contents in the numerical simulations.

MC (%)	*ε*′	*ε*″
3	1.9	0.15
6	2.25	0.21
9	2.58	0.29
12	2.94	0.39
15	3.31	0.49
18	3.76	0.6
21	4.23	0.72
24	4.75	0.84
27	5.17	0.97
30	5.44	1.08

**Table 2:** Linear fitting results between different reflection features and wood moisture content under simulation conditions.

Characteristics	*m*_1_	*m*_2_	*R* ^2^	*S*
Valley magnitude	−3.08	−53.02	0.97	0.32
Peak magnitude	22.61	143.97	0.97	0.04
Valley depth	2.73	−29.63	0.98	0.37

**Table 3:** Linear analysis results for valley depth and wood moisture content.

Species-Orientation	m_1_	m_2_	*R* ^2^	Sensitivity (dB/%)
Pine-x	6.06	−25.37	0.87	0.17
Pine-y	4.15	−21.13	0.77	0.24
Paulownia-x	7.12	−21.16	0.91	0.14
Paulownia-y	5.73	−24.30	0.98	0.17

**Table 4:** Independent-sample validation errors of the linear models.

Species-Orientation	RMSE (%)	MAE (%)
Pine-x	3.59	2.87
Pine-y	4.85	4.19
Paulownia-x	3.09	2.46
Paulownia-y	1.27	0.99

## Data Availability

The data presented in this study are available on request from the corresponding author.

## References

[1] Engelund E.T., Thygesen L.G., Svensson S., Hill C.A.S. (2013). A Critical Discussion of the Physics of Wood–Water Interactions. Wood Sci. Technol..

[2] Luimes R.A., Suiker A.S.J., Jorissen A.J.M., Van Duin P.H.J.C., Schellen H.L. (2018). Hygro-Mechanical Response of Oak Wood Cabinet Door Panels under Relative Humidity Fluctuations. Herit. Sci..

[3] Brandstätter F., Autengruber M., Lukacevic M., Füssl J. (2023). Prediction of Moisture-Induced Cracks in Wooden Cross Sections Using Finite Element Simulations. Wood Sci. Technol..

[4] Asare S., Stoescu M., Balmori L., Allena S., Kidando E., Owusu-Danquah J. (2024). Freeze-Thaw Effect on the Mechanical Properties of Different Wood Species: Impact of Moisture Content Variations, Microscopic Morphology, and Thermal Modification in a Selected Species. J. Infrastruct. Preserv Resil..

[5] Meyer L., Brischke C. (2015). Fungal Decay at Different Moisture Levels of Selected European-Grown Wood Species. Int. Biodeterior. Biodegrad..

[6] Fu Z., Chen J., Zhang Y., Xie F., Lu Y. (2023). Review on Wood Deformation and Cracking during Moisture Loss. Polymers.

[7] Běťák A., Zach J., Misák P., Vaněrek J. (2023). Comparison of Wood Moisture Meters Operating on Different Principles of Measurement. Buildings.

[8] Thybring E.E., Kymäläinen M., Rautkari L. (2018). Experimental Techniques for Characterising Water in Wood Covering the Range from Dry to Fully Water-Saturated. Wood Sci. Technol..

[9] Zhao X., Wang D., Guo P., Feng Q., Deng Y. (2025). Accuracy of Increment Core Method for Measuring Basic Wood Density and Moisture Content in Three Catalpa Species. Plants.

[10] (2009). Method for Determination of the Moisture Content of Wood.

[11] Mane S., Das N., Singh G., Cosh M., Dong Y. (2024). Advancements in Dielectric Soil Moisture Sensor Calibration: A Comprehensive Review of Methods and Techniques. Comput. Electron. Agric..

[12] Moron C., Garcia-Fuentevilla L., Garcia A., Moron A. (2016). Measurement of Moisture in Wood for Application in the Restoration of Old Buildings. Sensors.

[13] Paniagua V., Corrales J., Torres C., González-Rodrigo B. (2022). Influence of Timber Moisture Content on the Ultrasonic Wave Velocity Measurement of Tectona Grandis L.F. and Cupressus Lusitanica M. from Costa Rica. Forests.

[14] Gezici-Koç Ö., Erich S.J.F., Huinink H.P., Van Der Ven L.G.J., Adan O.C.G. (2017). Bound and Free Water Distribution in Wood during Water Uptake and Drying as Measured by 1D Magnetic Resonance Imaging. Cellulose.

[15] Hwang S.-W., Chung H., Lee T., Kwak H.W., Choi I.-G., Yeo H. (2023). Investigation of NIR Spectroscopy and Electrical Resistance-Based Approaches for Moisture Determination of Logging Residues and Sweet Sorghum. BioRes.

[16] Amaral E.A., Baliza L.F., dos Santos L.M., Shashiki A.T., Trugilho P.F., Hein P.R.G. (2023). Hydromechanical Behavior of Wood during Drying Studied by NIR Spectroscopy and Image Analysis. Holzforschung.

[17] Ismail R., Al-Mattarneh H., Malkawi A.B., Abuaddous M., Aljamal M., Trrad I. Prediction Moisture Content and Strength of Wood Using Free-Space Microwave Transmission Line NDT. Proceedings of the 2024 21st International Multi-Conference on Systems, Signals & Devices (SSD).

[18] Li J., Liu Z., Ma D., Chen S., Piao G. Detection of Wood Moisture Content Using Microwave Nondestructive Testing Method. Proceedings of the 2025 IEEE International Conference on Prognostics and Health Management (ICPHM).

[19] Wu Y., Zhao Z., Liu W., Zhang M. (2026). The Circuit Design and Modeling of a Wood Moisture Content Stratification Detection Instrument. Measurement.

[20] Suarez S., Sandhacker M., Logar L., Riegler M., Arminger B., Konnerth J., Gindl-Altmutter W., Tran A. (2026). Non-Invasive Method to Measure Bulk Electrical Resistivity of Spruce Wood—Influence of Moisture Content and Anatomical Direction. Wood Mater. Sci. Eng..

[21] Tamme V., Tamme H., Muiste P., Kangur A. (2025). A Novel Chrono-Potentiometry (CP) Method for Determining the Moisture Content of Wood Above the Fibre Saturation Point (FSP). Forests.

[22] Bogosanovic M., Al Anbuky A., Emms G.W. (2010). Overview and Comparison of Microwave Noncontact Wood Measurement Techniques. J. Wood Sci..

[23] D’Alvia L., Piuzzi E., Cataldo A., Del Prete Z. (2022). Permittivity-Based Water Content Calibration Measurement in Wood-Based Cultural Heritage: A Preliminary Study. Sensors.

[24] Schajer G.S., Orhan F.B. (2005). Microwave Non-Destructive Testing of Wood and Similar Orthotropic Materials. Subsurf. Sens. Technol. Appl..

[25] James W.L., Yen Y.-H., King R.J. (1985). A Microwave Method for Measuring Moisture Content, Density, and Grain Angle of Wood.

[26] Zhang V., Okumura S. (2000). Reduction of Multiple Reflection Influence in Microwave Free-Space Measurements. J. Microw. Power Electromagn. Energy.

[27] Johansson J., Hagman O., Fjellner B.-A. (2003). Predicting Moisture Content and Density Distribution of Scots Pine by Microwave Scanning of Sawn Timber. J. Wood Sci..

[28] Hansson L., Lundgren N., Antti A.L., Hagman O. (2005). Microwave Penetration in Wood Using Imaging Sensor. Measurement.

[29] Lundgren N., Hagman O., Johansson J. (2006). Predicting Moisture Content and Density Distribution of Scots Pine by Microwave Scanning of Sawn Timber II: Evaluation of Models Generated on a Pixel Level. J. Wood Sci..

[30] Aichholzer A., Schuberth C., Mayer H., Arthaber H. (2018). Microwave Testing of Moist and Oven-Dry Wood to Evaluate Grain Angle, Density, Moisture Content and the Dielectric Constant of Spruce from 8 GHz to 12 GHz. Eur. J. Wood Prod..

[31] Yu Y., Sun C., Wang L., Fan Y. (2025). A Dual-Parameters Microwave Measurement Approach for Spruce Moisture Content. Nondestruct. Test. Eval..

[32] Mai T.C., Razafindratsima S., Sbartaï Z.M., Demontoux F., Bos F. (2015). Non-Destructive Evaluation of Moisture Content of Wood Material at GPR Frequency. Constr. Build. Mater..

[33] Daassi-Gnaba H., Oussar Y., Merlan M., Ditchi T., Géron E., Holé S. (2017). Wood Moisture Content Prediction Using Feature Selection Techniques and a Kernel Method. Neurocomputing.

[34] Markel V.A. (2016). Introduction to the Maxwell Garnett Approximation: Tutorial. J. Opt. Soc. Am. A.

[35] Paz A., Thorin E., Topp C. (2011). Dielectric Mixing Models for Water Content Determination in Woody Biomass. Wood Sci. Technol..

[36] Rönnow D., Ottosson P., Andersson D. (2022). Microwave Complex Permittivity and Anisotropy of Conifer Wood Chips vs Moisture Content: Experiments and Modeling. J. Wood Sci..

[37] Gonçalves F.J.F., Pinto A.G.M., Mesquita R.C., Silva E.J., Brancaccio A. (2018). Free-Space Materials Characterization by Reflection and Transmission Measurements Using Frequency-by-Frequency and Multi-Frequency Algorithms. Electronics.

[38] Zhang H., Yang R., He Y., Foudazi A., Cheng L., Tian G. (2017). A Review of Microwave Thermography Nondestructive Testing and Evaluation. Sensors.

[39] Li M., Wang L., Zou X. (2024). Nondestructive Evaluation of Carbon Fiber Reinforced Polymer (CFRP)-Timber Interfacial Debonding Using Active Microwave Thermography (AMT). Constr. Build. Mater..

[40] Zou X., Li M., Lu X. (2026). Scanning Active Microwave Thermography for Detecting FRP-Timber Interfacial Defects. Eng. Struct..

[41] Wilcox L.M., Bonmarin M., Donnell K.M. (2024). Effect of Signal Modulation on Active Microwave Thermography. IEEE Trans. Instrum. Meas..

